# The influence of gender on clinical examination skills of medical students in Jordan: a cross-sectional study

**DOI:** 10.1186/s12909-020-02002-x

**Published:** 2020-03-31

**Authors:** Farnaz Sabet, Sohaib Zoghoul, Murad Alahmad, Heba Al Qudah

**Affiliations:** 1 Formerly at Department of Public Health and Community Medicine, Jordan University of Science and Technology, Irbid, Jordan; 2 Department of Diagnostic Radiology, Faculty of Medicine, Jordan University of Science and Technology, Irbid, Jordan; 3 Department of General Surgery and Urology, Faculty of Medicine, Jordan University of Science and Technology, Irbid, Jordan

**Keywords:** Medical students, Gender, Clinical skills, Intimate examinations

## Abstract

**Background:**

A graduating medical doctor is expected to be competent in physical examinations across all systems. The exploration of how gender affects the development of clinical skills has not been explored in an Arab context, despite cultural restrictions that make it more difficult for students and doctors to examine the opposite sex.

**Methods:**

A cross sectional survey was undertaken of graduating final year medical students in Northern Jordan. We asked about students’ perceptions regarding factors that may impact the development of clinical skills potentially related to gender, and asked about the frequency of examinations performed during their training for intimate and general physical examinations on all patients, as well as patients of the opposite sex. We also asked about the students’ confidence in performing the examinations (3-point Likert-scale). Comparison of male and female proportions was done using Chi square tests analysis.

**Results:**

One hundred eighty-eight final year students from 481 students (41%) completed the survey, 99 males and 89 females. The greatest factor given for impacting a student’s clinical examination of a patient of the opposite sex was cultural or religious traditions. Overall male students perform more clinical examinations than female students, with the odds of a male conducting more than 10 cardiovascular examinations on any patient compared to female students being 2.07 (1.13–3.79) and as high as 3.06 (1.53–6.18) for thyroid examinations. However, females were significantly more likely to examine male patients than vice versa (0.49 (0.27–0.88) for cardiovascular and 0.39 (0.21–0.71) for respiratory examinations). The gender division was more prominent for intimate examinations, with a lower odds of males conducting breast 0.11 (0.04–0.28) and vaginal examinations 0.22 (0.02–1.98) and more male students conducting prostate examinations OR 11.00 (1.39–87.03) and male genitalia examinations OR 16.31 (3.75–70.94). Overall a large proportion of students had never performed common intimate clinical examinations at all.

**Conclusions:**

In our context, clinical exposure to both intimate and general clinical examinations differs significantly between male and female students. A greater awareness and more research on the influence of gender on clinical skill attainment in conservative cultures is needed with appropriate adaption of clinical teaching.

**Trial registration:**

Non interventional thus not required.

## Background

One cornerstone of being a good doctor is competent clinical examinations, a standard followed by medical educators globally [1, 2]. Learning through examining real patients is a critical part of medical school training, and although there has been a rise in simulation learning, most would concur that learning through live patients is still required [3, 4]. Physical examination is part of the diagnostic process and can save considerable costs through decreased testing [5, 6] and its absence may adversely affect the doctor-patient relationship [7]. A graduating medical doctor is expected to be competent in all physical examinations, including intimate examinations [1] which are still an important component of clinical care [8]. Learning intimate clinical examinations, such as genital examinations has long been challenging for medical students due to their sensitive nature [9]. Various factors contribute to this such as anxiety and discomfort from students [10] and challenges finding patients who are willing to be examined [11].

However, what is considered to be an intimate examination differs in different cultures. In many settings in Jordan and other Middle Eastern countries, any touch between a male and female may be interpreted as intimate with a same sex doctor often requested by patients. This can be for abdominal, chest or thyroid examinations, as well as genital examinations. This perception and discomfort can be from both student and patient, with patient expectations to have private, quick and minimal clinical examinations. Students thus can find examining patients of the opposite sex in all system areas particularly challenging and this discomfort has been informally observed to persist after graduation by residency clinical training supervisors. However, clinical teaching in such a setting is often not adapted for such restrictions and delivered in a similar manner to a Western context, which may not be suitable, either for students or patients. Our understanding of this area is largely anecdotal, based on teaching and learning experiences. No previous work in the Arab region, to our knowledge, explores differences in clinical examination exposure and experience between female and male students.

One recent study conducted in Saudi Arabia in 2015 [12], explored student’s perception of intimate physical examination and sexual history taking and found most students never performed any intimate examinations and a large proportion had never taken a sexual history. Another Saudi Arabian study [13] looked at a similar question but in interns and with regards to sensitive examinations. This is the only study, to our knowledge, in the Arab region to look at both general examination competencies and intimate examinations with a view to capture different gender experiences among medical students.

There is an ongoing global change in the gender composition of medical schools [14] where the number of female students are increasing. In most Arab countries females constitute 40–60% of medical students [15], although most faculty is still male. This shifting composition of gender in medical schools will have an impact on teaching methods, medical culture and interaction with patients, particularly in countries where gender divisions are prominent. Gender has shown to affect medical practice [16, 17], medical student experience [18] and various elements of learning [19, 20] with most studies related to obstetric and gynaecology rotations [9, 16, 21, 22]. Thus, it is not unreasonable that in contexts with prominent gender divisions, gender would have far reaching implications on clinical learning experience which would then impact how faculty should teach.

In this study we surveyed final year male and female medical students in a Jordanian University about the frequency and self-efficacy in conducting both general and intimate examinations required to be performed by doctors, and analysed for any differences between male and female students.

## Methods

In order to begin to understand how gender influences medical student clinical examination exposure, the study surveyed a sample of final year medical students from Jordan University of Science and Technology (JUST) in Irbid, Jordan. The university has a strong reputation in the region, with approximately 3780 medical students in total, coming from more than 12 countries. During 2016, the year of the study, there were some 480 graduating medical students, with approximately 70% from Jordan. The curriculum is a six-year undergraduate one, with almost all admissions straight from secondary school. JUST medical graduates work not only in Jordan but across the entire Arab region, as well as internationally, such as in the United States and Europe. The female proportion of students is just over half.

Medical students and faculty staff are conscious of the restrictions placed on medical students in conducting various clinical examinations, both general and intimate. However, the various factors contributing to this and whether this translates to a significant difference between male and female student exposure to clinical examinations is unknown. We hope that such knowledge will enable faculty to better support medical student clinical examination learning in such contexts.

We aimed to determine the frequency and self-efficacy of final year male and female medical students in undertaking basic physical examinations, including cardiovascular, respiratory, gastrointestinal, and neurological examinations, as well as intimate physical examinations, such as pelvic and prostate examinations. Analysis was undertaken to determine any significant difference in examination skill exposure and self-efficacy between male and female students. Students were also asked about potential factors impacting their examination of the opposite sex, such as culture, supervisor support or personal factors.

Given the uniqueness of this context, a validated questionnaire was not available, so one was developed through an iterative process of brainstorming and piloting. A brainstorming session was held with a group of 20 medical students and a survey created. This was piloted on approximately 100 students, which was then followed by further discussion with a focus group of 10 medical students. Students who completed the questionnaire were asked for their feedback and we checked understanding of questions. The initial questionnaire aimed to look at all aspects of clinical medical teaching and to determine if frequency of exposure was different between males and females. There were questions about frequency of examinations and skills developed in areas as diverse as paediatrics, dermatology and ophthalmology. It also included an exhaustive list of factors that could potentially lead to decreased clinical exposure for students ranging from lack of confidence, to lack of privacy, cultural norms and feeling shy. Students’ feedback was that the questionnaire was too long, and as a result answers were not reliable. We felt that there was little to be gained by assessing every clinical examination especially when it was evident that common clinical examinations, such as cardiovascular exams, had a gender difference. The final questionnaire thus focusses on a few key clinical examinations and intimate examinations, and includes the top four factors identified in the pilot as factors that decreased clinical examination of the opposite sex. Questions on Grade Point Average were also removed as many students were not comfortable in providing their marks.

Language in the questionnaire was also reviewed, with terms clarified, for example, rectal examination was changed to prostate examination to make the examination clearly sex specific.

In addition to the number of times a student has performed an examination, their confidence in performing the examination on their own is also very important and may not always correlate with the number of times an examination is performed. Self-efficacy is a subjective assessment of one’s ability to perform a particular task successfully, and in theory increased confidence will be correlated with increased ability [23]. We devised a simple 3-point Likert scale to assess self-efficacy, with 1 - not confident at all to do the exam, 2 - need assistance or supervision to do the examination and 3 - able to do the examination on your own without supervision.

From the pilot it was determined that many students, both male and female, had no exposure to intimate examinations at all, and had performed few clinical examinations in general. We thus changed the survey with regards to frequency of examinations to include never having done the examination at all. It was also clear from the feedback that most students preferred an online survey, and so both online and paper options were offered, with the online survey offered through Survey Monkey (Palo Alto, CA) (A blank copy of the survey can be seen in Additional File 1).

The same 10 medical students who formed the focus group to refine the questionnaire, also publicised the project in various final year medical school lectures. They gave the link for the survey, as well as made paper copies available. It was difficult to engage final year students to complete even a short survey, as students face regular assessment in their last year. The group of volunteer students met regularly to review the number of completed surveys and undertook a weekly follow up campaign for a month through email and social media, as well as publicising in large lectures to increase the number of participants. All students opted to fill the survey online, which was easier to navigate and could be filled at their convenience. In order to prevent potential duplication of surveys, students were requested to provide their student identification number (ID), a unique 11-digit student identifier used for administrative purposes. During the initial brainstorm with students it was felt that this would provide adequate confidentiality as student IDs are not publicly available or known. Nonetheless, providing one’s student ID was made optional, and only 22 students chose not to do so. The inputs of these students were checked against all others and none were found to be duplicates.

Power was calculated using PS: Power and Sample Size calculations version 3.1.2 (Dupont and Plummer, Nashville, Tenn). Given no previous study exploring gender differences in clinical examination between male and female students in our setting, based on our clinical experience and the pilot, we took a conservative estimate that 50% of male students would have conducted male intimate examinations and 30% of females, and vice versa for female intimate examinations. For 80% power at 0.05 significance level using uncorrected chi square testing, a sample of 93 males and 93 females is required. Thus, our current sample size of 89 females and 99 males should be sufficiently powered for the purposes of our study.

Chi square tests were done on the questionnaire results using SPSS 18.0 (Chicago, SPSS Inc).

## Results

Out of 461 final year medical students 188 (41%) returned a completed questionnaire. The sample, although not random is closely representative of all final year medical students, across gender and nationality, except for fewer older medical students completing the study questionnaire than in the class cohort (Table 1). The ethnicities in our sample generally reflect the ethnicities of final year JUST medical students. This is important as those students who are not fluent in Arabic would potentially have language as a barrier to clinical examination. It is noted that the “other” ethnicity category is larger among all students, but this can be explained by the number of students who opted not to provide their nationality in the questionnaire.

**Table 1:** Demographics of students

*Variable*	*Sample students*		*All final year students*	
	*n = 188*	*Percent (%)*	*n = 461*	*Percent (%)*
Gender				
Female	89	47	203	44
Male	99	53	258	56
Nationality				
Jordanian	93	49.5	235	51
Malaysian	37	19.7	102	22.1
Saudi Arabian	11	5.8	34	7.4
Palestinian	4	2.1	9	2
Bahraini	6	3.2	14	3
Yemeni	3	1.6	8	1.7
Others	9	4.8	59	12.8
Missing	25	13.3	0	0
Age				
<20	0	0	0	0
20–25	175	93	349	75.7
>25	13	7	112	24.3

Students’ responses to various factors that may have affected their ability to conduct clinical examinations are listed in Table 2. Only 10 % of students have not been asked to leave a room during the clinical examination of another patient. However, in general, the majority of female and male students were only asked to leave the room either rarely or sometimes. In terms of perceptions of gender affecting a student’s clinical learning experience, it was males who felt that their gender gives them a greater positive learning experience compared to females (29% versus 20%). The majority of students surveyed, 64% females and 51% of males, felt that gender did not affect their learning experience at all.

**Table 2:** Factors affecting clinical examination as perceived by students

	All n (%)	Female n (%)	Male n (%)
Has a patient of the other sex refused to have you present during a clinical examination?			
Always	1 (1)	1 (1)	0 (0)
Never	19 (10)	9 (10)	10 (10)
Rarely	91 (48)	47 (53)	44 (44)
Sometimes	77 (41)	32 (36)	45 (46)
Total	188 (100)	89 (100)	99 (100)
How does your gender affect your learning experience?			
Does not at all	107 (57)	57 (64)	50 (51)
Negatively	34 (18)	14 (16)	20 (20)
Positively	47 (25)	18 (20)	29 (29)
Total	188 (100)	89 (100)	99 (100)
Which of the following have impacted your clinical examination of the other sex?			
Examining a patient from another sex may lead to misunderstanding	36 (11)	13 (9)	23 (13)
Examining a patient from another sex may make you shy/embarrassed	77 (23)	40 (27)	37 (20)
Lack of privacy of patients’ rooms in Jordan	68 (20)	27 (18)	41 (23)
Cultural and religious traditions	116 (35)	50 (33)	66 (36)
None of the above	35 (11)	20 (13)	15 (8)
Total	332 (100)	150 (100)	182 (100)
Do you feel supported by your supervisor to conduct intimate clinical examinations?			
Always	44 (23)	28 (32)	16 (16)
Never	18 (10)	3 (3)	15 (15)
Rarely	53 (28)	17 (19)	36 (36)
Sometimes	73 (39)	41 (46)	32 (32)
Total	188 (100)	89 (100)	99 (100)
Has a patient refused ever to give you a consent for doing an intimate clinical examination?			
No	44 (23)	18 (20)	26 (26)
Yes	144 (77)	71 (80)	73 (74)
Total	188 (100)	89 (100)	99 (100)
If you answered the last question with “yes” was the patient who refused of the other sex?			
No	40 (28)	18 (25.4)	22 (30)
Yes	103 (72)	52 (73.2)	51 (70)
Missing	1 (0)	1 (1.4)	0 (0)
Total	144 (100)	71 (100)	73 (100)

More than one answer could be given by students for reasons that could impact a student’s clinical examination of a patient of opposite sex. The greatest barrier in this regard were identified to be cultural and religious traditions, with 33% of females and 36% of males choosing this factor, followed by being shy or embarrassed. More than half of male students (51%) felt they were rarely or never supported to conduct intimate clinical examinations by their supervisors compared to 22% of females.

The results do indicate a large degree of patient discomfort and reluctance with having medical students conducting intimate examinations, with 77% of students surveyed having been refused permission for examination by the patient themselves, and most of these refusals being from patients of the opposite sex to the student.

We analysed the frequency of general physical examinations performed by students more than five times and more than 10 times (Table 3), to see if there was a difference between male and female student exposure to general physical examinations. For students who performed general examinations more than five times in a particular systems area, almost all our odds ratios are not significant with confidence intervals crossing one. However, once we analysed students with more clinical exposure, that is those who have performed particular clinical exams more than 10 times, it is males that dominate consistently, with approximately an odds ratio of 2 compared to females across the different main system areas. For example, the odds of a male doing more than 10 cardiovascular examinations compared to a female student is 2.07 (1.13–3.79) and as high as 3.06 (1.52–6.18) for a thyroid examination. Generally, male students do more clinical examinations than female students, but overall the frequency of clinical examination is not high, with a good proportion of students doing less than 10 basic clinical examinations during their clinical training. For example, only 71% of males and 55% of females performed a cardiovascular examination more than 10 times by the final year of their medical training.

**Table 3:** Frequency of general physical examinations performed throughout clinical training

*Physical Examination*	*Male n (%)*	*Female n (%)*	*OR (CI)*	*P value*
**Students who performed the examination > 5 times**				
Clinical exam performed on all patients				
Cardiovascular	89 (89.9)	75 (84.3)	1.66 (0.70–3.96)	0.248
Abdominal	96 (97)	84 (94.4)	1.91 (0.44–8.21)	0.480*
Respiratory	95 (96)	81 (91)	2.35 (0.68–8.08)	0.233*
Thyroid	61 (61.6)	46 (51.7)	1.50 (0.84–2.68)	0.170
Neurological	51 (51.5)	38 (42.7)	1.43 (0.80–2.54)	0.227
Clinical exam performed on patients of other sex				
Cardiovascular	46 (46.5)	57 (64)	0.49 (0.27–0.88)	**0.016**
Abdominal	57 (57.6)	67 (75.3)	0.45 (0.24–0.83)	**0.011**
Respiratory	52 (52.5)	66 (74.2)	0.39 (0.21–0.71)	**0.002**
Thyroid	43 (43.4)	24 (27)	2.08 (1.13–3.84)	**0.019**
Neurological	31 (31.3)	22 (24.7)	1.39 (0.73–2.64)	0.316
**Students who performed the examination > 10 times**				
Clinical exam performed on all patients				
Cardiovascular	71 (71)	48 (55)	2.07 (1.13–3.79)	**0.018**
Abdominal	88 (89)	72 (80)	1.89 (0.83–4.29)	0.124
Respiratory	80 (80)	62 (70)	1.83 (0.93–3.60)	0.076
Thyroid	36 (36.4)	14 (15.7)	3.06 (1.52–6.18)	**0.001**
Neurological	24 (24.2)	9 (10.1)	2.84 (1.24–6.51)	**0.011**
Clinical exam performed on patients of opposite sex				
Cardiovascular	30 (30)	35 (39.3)	0.67 (0.37–1.23)	0.194
Abdominal	39 (40)	46 (52)	0.61 (0.34–1.08)	0.091
Respiratory	36 (36)	42 (47)	0.64 (0.36–1.15)	0.132
Thyroid	30 (30.3)	9 (10.1)	3.87 (1.72–8.70)	**< 0.001**
Neurological	18 (18.2)	6 (6.7)	3.07 (1.16–8.14)	**0.019**

(*) used for values derived with Fisher’s exact test. Fisher’s exact test used when a cell in the table gave an expected number of frequencies fewer than 5

*P* values of < 0.05 written in bold

For exam frequency in patients of the opposite sex we see that for cardiovascular, abdominal and respiratory examination, more females were likely to examine the opposite sex, and for thyroid and neurological examinations the reverse was true, where more males were likely to examine female patients.

The proportion of students who performed a particular intimate examination more than five times, followed by the proportion of students who have never performed that examination is displayed in Table 4. As expected, more females conducted breast and vaginal examinations, and more males conducted prostate examinations and male genitalia examinations. However, it is surprising that males have performed more contraception counselling than females OR 2.73 (CI 1.14–6.52).

**Table 4:** Frequency of intimate examinations performed throughout clinical training

*Physical Examination*	*Male n (%)*	*Female n (%)*	*OR (CI)*	*P value*
**Students who performed the examination > 5 times**				
Male Genitalia Examination	27 (27.3)	2 (2.2)	16.31 (3.75–70.94)	< 0.001*
Prostate Exam	11 (11.1)	1 (1.1)	11.00 (1.39–87.03)	0.006*
Foley’s Catheter Insertion	3 (3)	0 (0)	NA	0.248*
Breast Examination	6 (6)	33 (37)	0.11 (0.04–0.28)	**< 0.001**
Vaginal Examination	1 (1)	4 (4.5)	0.22 (0.02–1.98)	0.191*
Pap Smear	2 (2)	0 (0)	NA	0.499*
Contraception Counselling	21 (21.2)	8 (8.9)	2.73 (1.14–6.52)	**0.021**
**Students who have never performed the examination**				
Male Genitalia Examination	14 (14.1)	45 (50.1)	0.16 (0.08–0.33)	**< 0.001**
Prostate Exam	51 (51.5)	75 (84.3)	0.20 (0.10–0.40)	**< 0.001**
Foley’s Catheter Insertion	74 (74.7)	76 (85.4)	0.51 (0.24–1.06)	0.07
Breast Examination	44 (44)	1 (1.1)	70.4 (9.43–525.7)	**< 0.001**
Vaginal Examination	93 (93.9)	63 (70.9)	6.40 (2.49–16.43)	**< 0.001**
Pap Smear	94 (94.9)	84 (94.4)	1.12 (0.31–4.00)	0.863*
Contraception Counselling	35 (35.4)	25 (28)	1.40 (0.75–2.60)	0.286

(*) used for values derived with Fisher’s exact test. Fisher’s exact test used when a cell in the table gave an expected number of frequency fewer than 5

*P* values of < 0.05 written in bold

Many students have never undertaken particular intimate examinations at all. Almost all students have never conducted a vaginal examination and 75% of females and 51% of males had never conducted a prostate examination. Only a very small number of students have performed any intimate examination more than five times by the final year of medical school training. No female student surveyed conducted more than five pap smears during her training and only three male students inserted a Foley’s catheter more than five times (despite catheter insertion being a doctor’s responsibility in Jordan).

We asked students if they are confident doing the specified clinical examinations on their own without supervision (Table 5). There was no notable difference between male and female students. This was not the case with regards to intimate physical examinations (Table 5), where compared to the numbers of students who had performed the examination more than five times, there were a greater number of students who were confident performing the examination. 33% of males were confident in performing a male genital examination, yet only 27% of males have performed the examination more than five times. 49% of males and 37% of females are confident to do contraception counselling on their own, with only 21% of males and 8% of females having performed contraception counselling more than five times. Very few students graduate being confident in performing intimate clinical examinations. Only 7% of females and 19% of male students surveyed are confident to do a digital rectal examination and only a handful are confident to do a vaginal examination or a pap smear.

**Table 5:** Number of students confident to perform examination without supervision

*Physical Examination*	*Male n (%)*	*Female n (%)*	*OR (CI)*	*P value*
Cardiovascular	69 (69.7)	57 (64)	1.29 (0.70–2.38)	0.410
Abdominal	83 (83.8)	79 (88.8)	0.66 (0.28–1.53)	0.329
Respiratory	77 (77.8)	74 (83.1)	0.71 (0.34–1.47)	0.355
Thyroid	73 (73.7)	64 (71.9)	1.10 (0.58–2.09)	0.778
Neurological	53 (53.5)	36 (40.4)	1.70 (0.95–3.03)	0.073
**Intimate Physical Examination**				
Male Genitalia Examination	33 (33.3)	6 (6.7)	6.92 (2.74–17.50)	**< 0.001**
Digital Rectal Examination	19 (19.2)	7 (7.9)	2.78 (1.11–7.00)	**0.025**
Foley’s Catheter Insertion	13 (13.1)	3 (3.4)	4.33 (1.19–15.75)	**0.019**
Breast Examination	20 (20.2)	51 (57.3)	0.19 (0.10–0.36)	**< 0.001**
Vaginal Examination	7 (7)	6 (6.7)	1.05 (0.34–3.26)	0.929
Pap Smear	5 (5)	3 (3.3)	1.53 (0.35–6.57)	0.724*
Contraception Counselling	49 (49.5)	37 (41.6)	1.38 (0.77–2.45)	0.276

(*) used for values derived with Fisher’s exact test. Fisher’s exact test used when a cell in the table gave an expected number of frequency fewer than 5. *P* values of < 0.05 written in bold

## Discussion

This study is the first, to our knowledge, to explore how gender may impact the learning of clinical examination skills in medical students in a conservative setting. Clinical examination skills remain a core component of clinical care and understanding the limitations of student exposure to conducting examinations is important.

The study suggests that in general, doing clinical examinations in this cohort of students is limited. Not all students have performed basic clinical examinations such as cardiovascular or respiratory examinations more than five times by the final year of medical school, even fewer have conducted more than 10 examinations on patients. As expected, this number is much less for intimate examinations. Only a very small number of students had performed any intimate examination more than five times, and a large proportion of students have never performed certain intimate examinations. However, as this study also assessed frequency of general clinical examinations, the low number of intimate examinations performed can also be attributed to general overall limited exposure to performing clinical examinations by students on patients. One reason for this may be an increasing number of student admissions, and larger groups of students in bedside tutorials which limits student exposure to clinical skill learning. This coupled with a lack of a formal supervision process for clinical examination skill learning, such as a log book is anecdotally associated with decreased clinical skill confidence in students. Other reasons include an often reported decline in the clinical skills of medical students and residents and a lack of acknowledgement of their importance by students [24]. Although frequency of performing examinations is insufficient to assess performance, our study nonetheless provides a simple gauge for medical schools to assess if teaching is providing sufficient exposure or not.

Gender appears to affect students’ clinical experience in multiple ways, despite more than half of surveyed students stating that gender had no impact on their clinical learning. During the pilot we found that most students did not recognise the role that gender played in their clinical exposure and our study confirmed this. The large gender divide in performing intimate examinations is expected, but the gender divide was also present in the examination of other systems. When observing the number of clinical examinations performed on patients of the opposite sex in particular, we find that the majority of patients who refused students to clinically examine them were of the other sex. We also see that female students were able to perform cardiovascular, abdominal and respiratory examinations more frequently in opposite sex patients. This is likely due to male patients being more comfortable about exposure of their chest or abdominal area compared to female patients, and students more comfortable in requesting exposure. This gender differentiation was not seen with thyroid and neurological examination, which require less exposure. Moreover, thyroid pathology is more common in females and thus the opportunity for male students to conduct a thyroid exam in a female is likely higher. Students mentioned that the greatest barrier to examining patients of the opposite sex were cultural and religious traditions, followed by being shy or embarrassed, and this correlates with students being able to complete more examinations that require less exposure in the opposite sex.

Although we find that female students are less restricted in performing clinical examinations, overall more males than females felt that gender positively impacts their learning experience. Indeed, when looking at students who performed more than 10 clinical examinations in a system area, males consistently outperform females, with an odds ratio of about 2 across different main system areas. There may be multiple factors contributing to this. Firstly, most of the teaching staff is male. At the time of the study only 15.2% of the entire faculty was female, and only 2.3% of full professors were females. Thus, role models and appropriate mentorship to female students may be limited. Although in our study, less males than females felt supported by supervisors to conduct intimate examinations, nonetheless, overall mentorship and opportunities may be higher for males. Secondly, the patient population may still be more comfortable with care from the traditional perception of a doctor, who is male. Thirdly, there may be an overall organizational culture that favours the male physician or student especially in clinical settings, as is the case in other contexts [18]. This study suggests that there is a complex, likely multifactorial, interplay between gender in this setting and clinical learning, that will warrant further research. There is also a need to better support female students to develop clinical skills, as identified elsewhere [18], with for example concerted efforts to recruit female faculty and greater awareness around gender bias.

Conversely, in the specific area of conducting intimate examinations males can be subject to gender discrimination with tutors selecting female students more often and males feeling less encouraged to conduct intimate examinations [21, 25]. This was confirmed in our study with more than half of male students (51%) feeling they were rarely or never supported to conduct intimate clinical examinations by their supervisors compared to 22% of females. In this regard, male students will require greater support across a few areas from supervisor encouragement to patient acceptance to ensure that they also develop a full clinical skill set.

The limited number of intimate examinations performed by students during their training is concerning. A large proportion of both sexes have never done certain intimate examinations, with over 90% of males and 70% of females never performing a vaginal examination during their training. Yet, the issue does not seem to lie only with conducting clinical examinations, as more than a quarter of students had never done any contraception counselling. It may be any discussion or examination around sexual and reproductive health is difficult for students to engage in.

The study has a number of limitations. Firstly, the questionnaire used, although developed with a great deal of student input and was piloted, was not validated. Students were final year students and were estimating the number of examinations they had undertaken during their training based on recollection, so subject to recall bias, and did not have a log book or record to refer to.

Our sample was only 42% of all final year students, and though generally representative of all students in key indicators such as gender and nationality, we do not know if this sample was representative in terms of clinical exposure. They could have been students who were more eager, or less committed to learning clinical skills. Nonetheless, they do give important insights that confirm student and faculty experience.

Measuring competence in clinical examinations is a challenge, and ideally requires assessing the actual action on a real patient (see Miller’s triangle [26]). We did not have resources to do this for the number of clinical examinations and students being surveyed, and would have great difficulty undertaking observed intimate examinations in our context. Self-efficacy itself is problematic, and there are many studies that show that subjective self-assessment does not always correlate with objectively measured performance [23, 27, 28]. Further studies with more resources may be able to actual observe and assess clinical skill.

The results may have been biased as a multivariate approach was not used in data analysis to control for potential confounding factors. Qualitative studies would be helpful to explore some of the complex factors affecting the interplay of gender in clinical skill learning int this setting, which are currently unknown.

Despite the limitations, the study does provide important insights into areas that require improvement in medical teaching in such a context. It is clear that clinical examination skills may need to be taught in different and novel ways. Intimate examinations can be taught through lectures, videos, models, simulation or standardised patients. Jha and colleagues conducted a systematic review on patient involvement in teaching and assessing intimate examinations [4]. They found that involving a patient, whether real or simulated, led to improved clinical performance and reduced anxiety in students and not only developed clinical examination skills, but the interpersonal and communication skills required in conducting intimate examinations, and enhanced student awareness of patient anxiety around the examination [4]. In our setting, recruiting simulated patients is highly challenging, if not impossible. A realistic and alternative method of teaching needs to be explored.

A systematic review and meta-analysis from 2013 on simulation training for breast and pelvic examinations, found that simulation training compared to no intervention was associated with moderate to large improvement in effects on skills outcomes [29]. This was enhanced when dynamic models were used that could provide feedback for breast examinations and an electronic model with enhanced feedback for pelvic examinations. Further research was suggested to explore instructional approaches that could enhance skill development [29]. Despite the benefit of simulation compared to no intervention, interaction with a human is more likely to actually translate into good bedside skills [30] and enable educators to properly assess clinical skills [31]. However, all the studies for both of these two systematic reviews either came from North America or Europe.

As seen in this study, gender not only affects intimate examination learning, but other systems as well, in particular examinations that require more exposure such as of cardiovascular and respiratory systems. Using simulation for such systems may also be helpful, but it is also likely insufficient. For example, learning cardiovascular examinations through simulation, including with audio recordings does not necessarily result in improved practical clinical skills [32]. There is strong evidence that additional clinical skills and assessment are needed in medical school training [33].

In this context we also need to consider if medicine should be practiced the same way as in a Western context. There are universities in the region that segregate students based on sex [34] with different instructors for males and females, and they may have further discrepancies in clinical skill attainment between the two sexes. The gender divide may well translate into most clinical settings, but this is unknown. However, if these students then practice in other settings, where there is no gender divide in clinical care, then assessment of a full skill set may be required.

## Conclusions

In a conservative setting, our study found that clinical examination and exposure, both general and intimate, differs between female and male students. There are numerous areas where gender influences medical student clinical learning, including clinical exposure, supervisor support and patient willingness and consent. Male students felt that overall being male lent itself to a more positive learning experience. Nonetheless the role of gender in clinical experience is minimised, both by students in this study, and in general.

In every setting there is a need to be conscious about the needs of all students to ensure that all graduates have basic competencies. In our context, where gender divisions are more prominent, simply adopting standards and curriculums from other settings may mean that students are left deficient in exposure and skills. More effort is needed, particularly with the changes resulting from the ongoing feminisation [35] of the medical workforce, to ensure that all students are developing adequate skills and providing appropriate and full patient care.

## Supplementary information

**Additional file 1.** Blank copy of the survey.

## Data Availability

The datasets used and/or analysed during the current study are available from the corresponding author on reasonable request.

## Abbreviations

CI: Confidence Interval
OR: Odds Ratio
