# Supplementary material for: The influence of gender on clinical examination skills of medical students in Jordan: a cross-sectional study
Source: BMC Med Educ. 2020 Mar 31;20:98. doi: 10.1186/s12909-020-02002-x (PMC7110726; doi:10.1186/s12909-020-02002-x)
Supplement: Supplementary file 1 — Additional file 1. Blank copy of the survey. [file 12909_2020_2002_MOESM1_ESM.pdf]

The Influence of Gender on Clinical Examination Skills

Thank you for agreeing to complete this survey. All information collected will be used only for research purposes and your answers will be anonymous and confidential.

This survey is for final year medical students. There are 13 questions in total.

1. what is your age?

[ ]

2. what is your gender?

- ◯ Male
- ◯ Female

3. What is your nationality?

[ ]

4. What is your student number (ID)? (eg: 20110010017)

[ ]

5. Has a patient of the opposite sex refused to have your presence in the room /clinic during the clinical examination?

- ◯ never
- ◯ rarely
- ◯ sometimes
- ◯ always

6. How does your gender (male/female) affect your learning experience?

- ◯ positively
- ◯ negatively
- ◯ it does not affect my learning experience at all

7. Which of the following have impacted (affected) your clinical experience when doing examinations on the opposite sex?

*you can choose more than one answer

- [ ] examining a patient from another sex may lead to misunderstanding
- [ ] examining a patient from another sex may make you shy
- [ ] patient's rooms in Jordan lack privacy for clinical examinations
- [ ] cultural and religious traditions

8. Do you feel supported by your supervisor (consultant) to conduct intimate clinical examinations?
*intimate: examinations include breast, genitalia and rectal examinations.

- ◯ never
- ◯ rarely
- ◯ sometimes
- ◯ always

9. Has a patient refused to give you a consent for doing an intimate clinical examination?

- ◯ yes
- ◯ no

10. If you answered the last question with "yes" was the patient who refused of the opposite sex?

- ◯ yes
- ◯ no

11. How many times have you done the below listed examinations throughout medical school approximately?

How many of them were on patients of the opposite sex?

And how confident are you to do the examination now? Please use the numbers below to answer.

*frequency:
1) 0 times
2) 1-5 times
3) 6-10 times
4)more than 10

*confidence:
1) not confident at all
2) need assistance/supervision
3) confident to do it on own

	frequency in all patients	frequency in opposite sex	confidence
cardiovascular examination			
abdominal examination			
respiratory examination			
thyroid examination			
neurological examination			

12. How many times have you done the below listed intimate male examinations throughout medical school approximately?

How many of them were on patients of the opposite sex?

And how confident are you to do the examination now? Please use the numbers below to answer.

*frequency:
1) 0 times
2) 1-5 times
3) 6-10 times
4) more than 10 times

*confidence:
1) not confident at all
2) need assistance/supervision
3) confident to do it on own

	frequency in all patients	frequency in opposite sex	confidence
male genitalia examination			
digital rectal examination and prostate examination			
foley's catheter insertion			

13. How many times have you done the below listed intimate female examinations throughout medical school approximately?

How many of them were on patients of the opposite sex?

And how confident are you to do the examination now? Please use the numbers below to answer.

*frequency:

1) 0 times
2) 1-5 times
3) 6-10 times
4) more than 10 times

*confidence:

1) not confident at all
2) need assistance/supervision
3) confident to do it on own

	frequency in all patients	frequency in opposite sex	confidence
breast examination			
vaginal examination			
digital rectal examination			
pap smear			
contraception counselling			

Thank you for your participation.
